# A review on 3D printing functional brain model

**DOI:** 10.1063/5.0074631

**Published:** 2022-02-03

**Authors:** Roya Samanipour, Hamed Tahmooressi, Hojatollah Rezaei Nejad, Minoru Hirano, Su-Royn Shin, Mina Hoorfar

**Affiliations:** 1 Department of Mechanical Engineering, University of British Columbia, Kelowna, British Columbia V1V 1V7, Canada; 2 Department of Medicine, Brigham and Women’s Hospital, Harvard Medical School, Boston, Massachusetts 02139, USA; 3 Department of Electrical and Computer Engineering, Tufts University, 161 College Avenue, Medford, Massachusetts 02155, USA; 4 Future Vehicle Research Department, Toyota Research Institute North America, Toyota Motor North America, Inc., 1555 Woodridge Ave., Ann Arbor, Michigan 48105, USA; 5 Faculty of Engineering, University of Victoria, Victoria, British Columbia V8W 2Y2, Canada

## Abstract

Modern neuroscience increasingly relies on 3D models to study neural circuitry, nerve regeneration, and neural disease. Several different biofabrication approaches have been explored to create 3D neural tissue model structures. Among them, 3D bioprinting has shown to have great potential to emerge as a high-throughput/high precision biofabrication strategy that can address the growing need for 3D neural models. Here, we have reviewed the design principles for neural tissue engineering. The main challenge to adapt printing technologies for biofabrication of neural tissue models is the development of neural bioink, i.e., a biomaterial with printability and gelation properties and also suitable for neural tissue culture. This review shines light on a vast range of biomaterials as well as the fundamentals of 3D neural tissue printing. Also, advances in 3D bioprinting technologies are reviewed especially for bioprinted neural models. Finally, the techniques used to evaluate the fabricated 2D and 3D neural models are discussed and compared in terms of feasibility and functionality.

## I. INTRODUCTION

Progress in understanding the human nervous system and elucidating the mechanisms of a variety of mental disorders is greatly limited due to the restricted access to actual functioning of human brain tissues. As a result, 3D *in vitro* neural models have recently received significant ever-growing attention. The modern neuroscience mainstream increasingly relies on 3D models to study neural circuitry, neural regeneration, and diseases.^1^ Functional 3D neural tissue models can provide insight into brain development, exploration of new therapeutic solutions, and cost-effective drug discovery investigations. In the long term, 3D *in vitro* neural models will potentially represent the human neural system better and can be used more often in regenerative medicine.^2,3^

Here, 3D *in vitro* models are categorized into cell-based models (e.g., spheroids and organoids) and engineered models (e.g., scaffold-based, microfluidics, etc.) Spheroids can be seen as non-adherent 3D cell cultures where their degree of heterogeneity is a function of their initial cell population. Organoids have a higher order of assembly due to the presence of a special matrix (e.g., Matrigel) and spatial cues, which leads to the formation of organ-like structures. In contrast, scaffold-based models use fully defined constructs as a scaffold for controlled cell growth. Microfluidic models rely on manipulation of fluid flow to define the culture environment. Perfusion flow, spatial control over cell patterning, and co-culture of cells can be achieved by microfluidic platforms.^4^

**FIG. 1.**
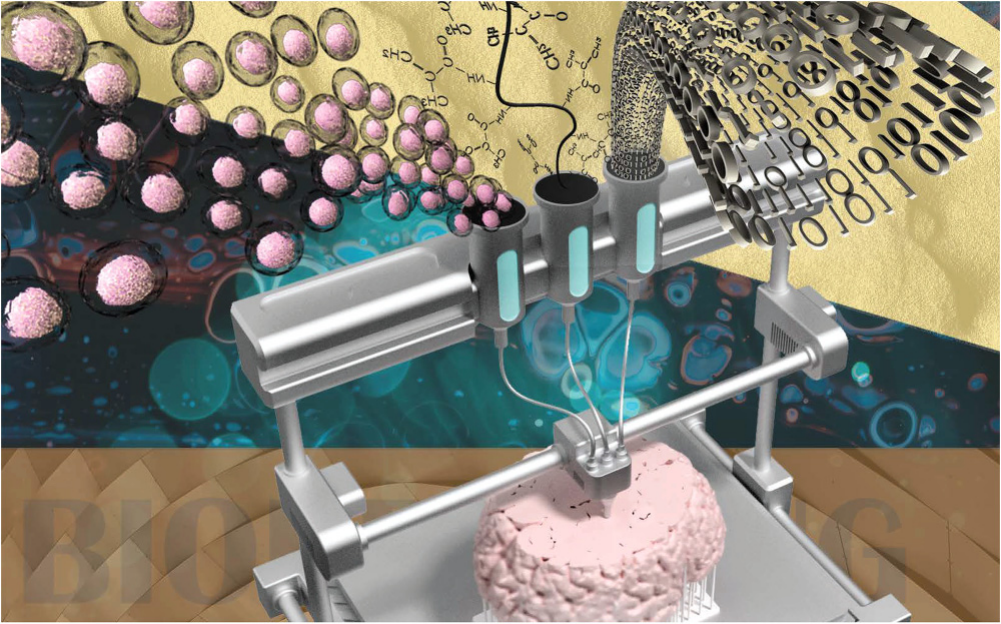
Schematic representation of 3D bioprinting of neural tissues. Biomaterial, living cells, and numerical control are the main components of bioprinting.

Cell-based models have a better performance when it comes to the imitation of the early developmental stages. On the other hand, engineered models are aimed to control the composition of the scaffold material and cell organization, which leads to more controlled and consistent tissue-like constructs. Regardless of the type of the model (cell-based or engineered), a user-controlled and replicable approach to mimic the native neural environment is of great importance. 3D bioprinting emerged to address the lack of flexibility in spatially positioning the cells within the desired architecture.^5^ In essence, 3D printing is an automated high-throughput platform (with exceptional versatility in cell positioning) that provides relatively fast fabrication of tissue constructs with complex 3D topologies (Fig. 1). 3D bioprinting comes in a variety of forms (including inkjet, extrusion, and stereolithography printing) with different capabilities and limitations. The main challenge in adapting 3D printing technologies for biofabrication of neural tissue models is to develop the neural bioink, i.e., a biomaterial or composition of multiple biomaterials with special properties, such as printability, gelation, and suitability for neural tissue culture.^6^ Considering the importance of 3D bioprinting in the development of 3D *in vitro* neural models, this review paper provides a thorough outlook of fundamentals and principles of 3D neural tissue models. It also covers advances in different 3D bioprinting platforms. Finally, functionality of neural tissue models for a vast range of neural biomaterials and bioinks is discussed.

## II. 3D BRAIN MODEL FUNDAMENTALS

### A. 3D brain model design factor

Human brain is a complex 3D structure of neural cells embedded in a matrix known as an extra cellular matrix (ECM). The physiochemical properties of ECM play a crucial role in cell–cell and cell–environment communications. Inevitably, presence of the third dimension is essential to reach such a high level of functionality, while simplifying such a composition into 2D models causes aberration of neural phenotype.^7^ Additionally, there are numerous biological/biochemical/biophysical factors at a cellular and environmental level that need to be considered in building an organ *in vitro*. Since fabricating the entire organ is not possible in most cases, a more practical approach is to generate a 3D *in vitro* tissue model that recapitulates the essential elements of the organ, a functional unit of an organ.^8^ These 3D *in vitro* models should be evaluated based on their ability in mimicking the bio-functionality of the native tissue. Advances in bioprinting technologies can enhance the physical properties of 3D tissue models to regenerate the physiology of the native tissue in order to improve the accuracy of the models.^9^ Here, we describe the fundamental features of the neural tissue as a guideline for designing neural tissue constructs. In essence, the neural system contains various cell types communicating in a synchronized harmony. The central nervous system (CNS) itself has around 86 × 10^9^ neurons and 85 × 10^9^ non-neural cells, known as glial cells.^10^ Glial cells can also be categorized into astrocytes, microglia, oligodendrocyte, pericytes, and ependymal cells.^8^ Therefore, a dense diversity of different cell types should be considered for a 3D *in vitro* model to enable appropriate cell–cell interaction and cell phenotype.

**FIG. 2.**
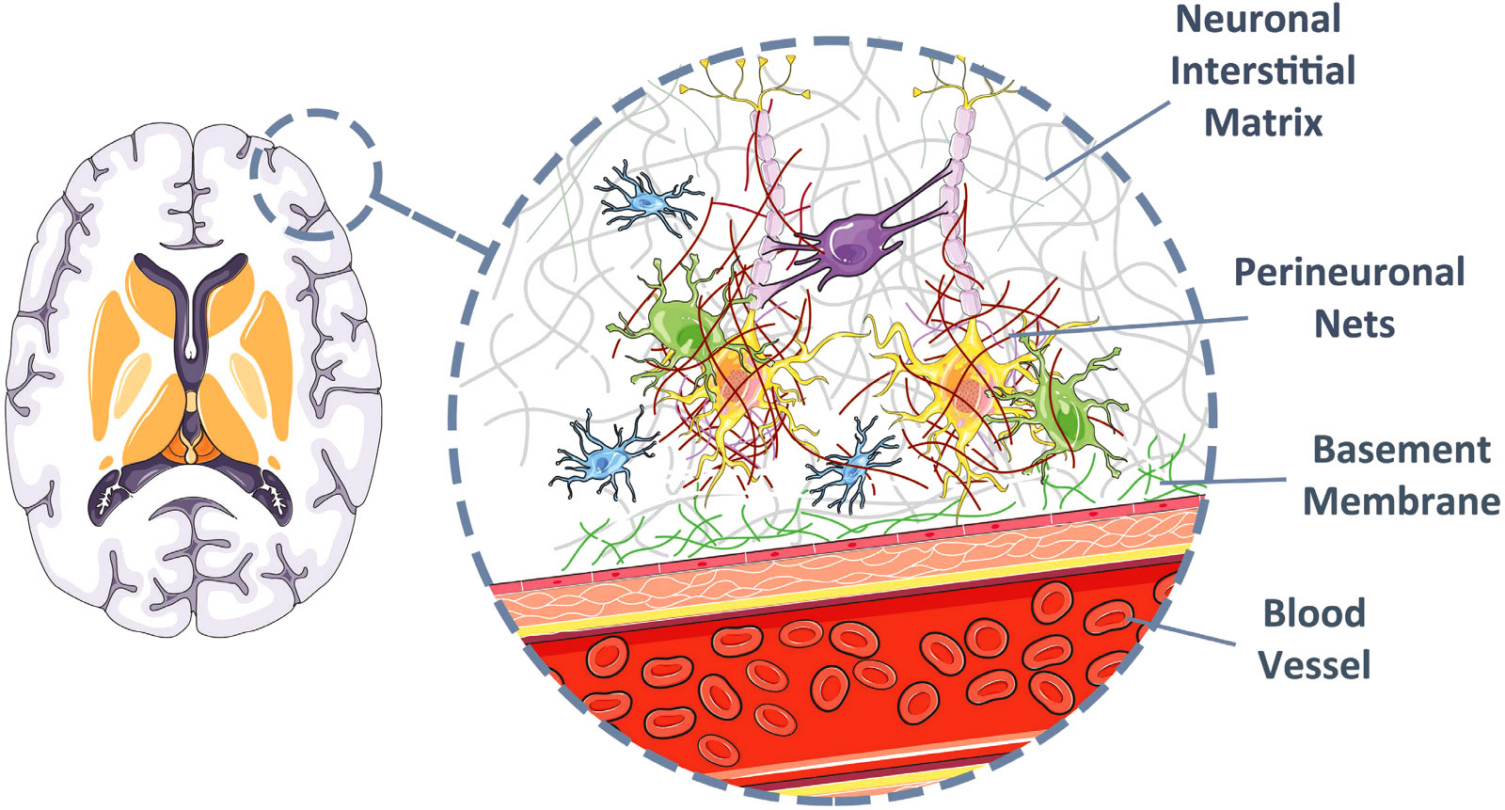
Cellular structure of the brain tissue. The cellular structure of the brain tissue ECM can be divided into three main components: basement membrane, perineuronal nets, and neuronal interstitial matrix. Image created under a Creative Commons Attribution (CC BY) license (smart.servier.com).^15^

Prior to studying the design factors and characteristics of the brain ECM, it is paramount to gain an overall picture of the complexities and characteristics of the brain cellular structure (Fig. 2). Understanding the cell-level hierarchy of the brain tissue is an important step toward printing a successful model of the brain tissue. The cell type diversity, mechanical properties of the surrounding environment, and the chemical interactions between the cells and the environment together represent a complex living matrix. The brain's extracellular matrix (ECM) is a support environment for the neurons and glia that regulates their migration, proliferation, and synaptic integrations.^11^ The macromolecular scale structure of the brain tissue ECM can be divided into three main components. The first component, the basement membrane, covers the cerebral vasculature, which together with endothelial cells, pericytes, and astrocytes forms the blood–brain barrier (BBB).^12^ The basement membrane works as a regulator between endothelial cells and brain parenchymal cells. The ECM in this layer is made of four main proteins, including collagen IV, laminin, nidogen, and perlecan.

Passing through the basement membrane, neurons are embedded inside a special lattice that regulates the synaptic interactions by adjusting the dendrite–soma distances.^13^ The structure of the ECM at the perineuronal net, in contrast to the rest of the body, is less fibrous. This gives the neurons more flexibility and provides a platform to create potential neuronal pathways. The perineuronal net is mostly made of proteoglycans, tenascin R, and link proteins.^14^ The high polarity of the proteoglycans attracts water molecules and creates a softer tissue, in contrast to more fibrous ECM. The last component of the brain ECM structure is the interstitial that connects the neurons and the vasculature.^13^ It consists of the ECM molecules diffused in the parenchyma.

Another major factor for designing neural constructs is the composition of ECM. The neural system, in fact, has a unique ECM mainly made of proteoglycans of tenascin, hyaluronan, and lectican.^16^ Interestingly, a component, such as a laminin, fibronectin, and collagen, are presented in a much lower quantity as compared to other parts of the human body.^8^ ECM of the neural system also contains a high number of soluble factors (including growth factors, chemokines, and cytokines).^17^ Neural development and circuit formation, including the dendrite and axonal outgrowth, are targeting functions for finely tuned growth factor concentrations. Consequently, a key component to be included in the *in vitro* 3D models is to establish such a concentration distribution to facilitate nerve regeneration.^18–21^

Aside from the neural composition, neural ECM may also regulate cell behavior through its physical and mechanical properties. Neural ECM has distinctive biophysical properties, such as low elastic modulus and large porosity, as compared to other tissues, such as heart, cartilage, and bones.^7^ In particular, the elastic modulus of the brain tissue is approximately 110 Pa for neonatal and less than 1 kPa for adults.^7^ It is also known that matrix stiffness has a significant effect on neural cell behavior and morphology.^22–24^ For example, neural stem cells are more likely to differentiate into glial cells when their surrounding matrix has Young's modulus higher than 1 kPa. Softer ECM (100–500 Pa), however, tends to promote cell migration and differentiation into neurons.^25–28^ In another example, mesenchymal stem cells undergo neural differentiation (express neural gene) on soft substrates (1 kPa), whereas on stiffer matrices (10 kPa), they tend to differentiate into glial lineage.^29^ Similarly, ECM porosity can greatly affect cell behavior and cell metabolism.^29^ Large pore sizes (more than 1000 *μ*m) facilitate appropriate nutrient exchange, while ECM with porosity similar to the native tissue results in a better cell migration.^30^ Neural cells have also shown to respond to microstructure and geometrical cues.^31–35^ Such cues have proven to improve cell viability,^36^ migration,^37^ proliferation,^25^ and differentiation.^38^ Topological cues have also shown to facilitate neurite outgrowth.^23,39–41^ The recapitulation of parameters, such as biochemical and physical gradients, biocompatibility, porosity, mechanical property, and multiple cell types, is a crucial step in reconstruction of a successful neural tissue.

After determining the key factors for 3D neural model design, the next step is to find the right fabrication technique. Different techniques have been used to fabricate 3D neural models, including a hanging drop platform for self-organized models (e.g., spheroid used as neural network building blocks^42^ and human midbrain organoids^43^) and microfluidics (e.g., blood–brain barrier^44^). Although these techniques have enhanced our understanding of neural tissue development, the lack of precise spatial configuration of different cell types is still a challenge. Composite cellular architecture is a very crucial parameter for signaling the physiologically relevant cues that leads to generation of functional tissue models. Moreover, these methods cannot be considered a high-throughput technique.^8^ Recently, bioprinting has shown potential in fabricating heterogeneous tissue models with great consistency.^45^ Using such techniques, cells are mixed with chemical cues and relevant biomaterial printed in a 3D structure. Therefore, the cell can be organized spatially in an engineered construct.^46^ In Sec. III, we describe the bioprinting techniques as well as their advantages and disadvantages.

### B. Types of neural cells

Three major sources of cells available for the development of the neural tissue models include primary neural cells, stem cells, and immortalized cell lines. Brain derived primary cells, collected from dissociated tissues, are usually used directly after isolation at passage zero.^7^ Immortalized cell lines, however, can be passaged for a higher number of splits in the culture. Aside from these types, stem cells are also a great source of cells for neural cultures. These cells mainly include embryonic-derived stem cells, neural progenitors, or induced pluripotent stem cell (iPSC)-derived neurons.^47,48^ A Human neural tissues are not a readily available source of primary cells for *in vitro* cultures.^49^ Moreover, most adult neural cells have limited regenerative capabilities, and therefore, it is hard to expand *in vitro*. As 3D cultures require very high seeding densities, other mammalians (such as rat and mice) were considered to develop neural models.^7,50^ Although the cultured cells from rodents are readily available and used in 3D neural tissues, they have significant differences both in organization and function in comparison with human cells (e.g., different ratios of neurons to glia cells).^51–53^ In addition, primary neurons can lose functionality during the isolation process. Primary cells also reach senescence and become quiescent after a limited number of splits and, therefore, are not suitable for long run *in vitro* neural cultures.^7^ To address the challenges faced with primary cells, immortalized cell lines are vastly used in neural cell cultures. Neural cell lines could be achieved from brain tumors (e.g., by genetically modifying healthy cells or neuroblastoma).^54,55^ Some of the widely used mammalian cell lines for deriving neural cells through *in vitro* differentiation are pc12 (rat source) and p19 (mice source).^55^ Although neural cell lines are helpful for studying neuronal process and *in vitro* differentiation, they may not have the biological and biochemical characteristics as compared to the native tissue. For these reasons, the usage of neurons derived from stem cells and iPSCs of a human origin has been considered.^7^ For instance, it has been shown that pluripotent stem cells provide flexibility for differentiation into neurons, astrocytes, and oligodendrocytes.^7^ Stem cells can also be passaged to high numbers, as high cell density seeding is required for neuron culture. To create neural cell types, the most encouraging sources are mesenchymal stem cells (MSCs) derived from adipose tissue, bone marrow, skin, and umbilical cord. Many other tissue sources have also been successfully utilized.^7^ Some research indicates that adipose tissue-derived-MSCs have more potential to differentiate to neural cells with a high-level expression of neural markers and proliferation capacity. Embryonic stem cells (ESCs) and iPSCs from many types of somatic cells can also differentiate into neural cells).^56,57^

## III. 3D BIOPRINTING TECHNOLOGIES FOR THE BRAIN

**FIG. 3.**
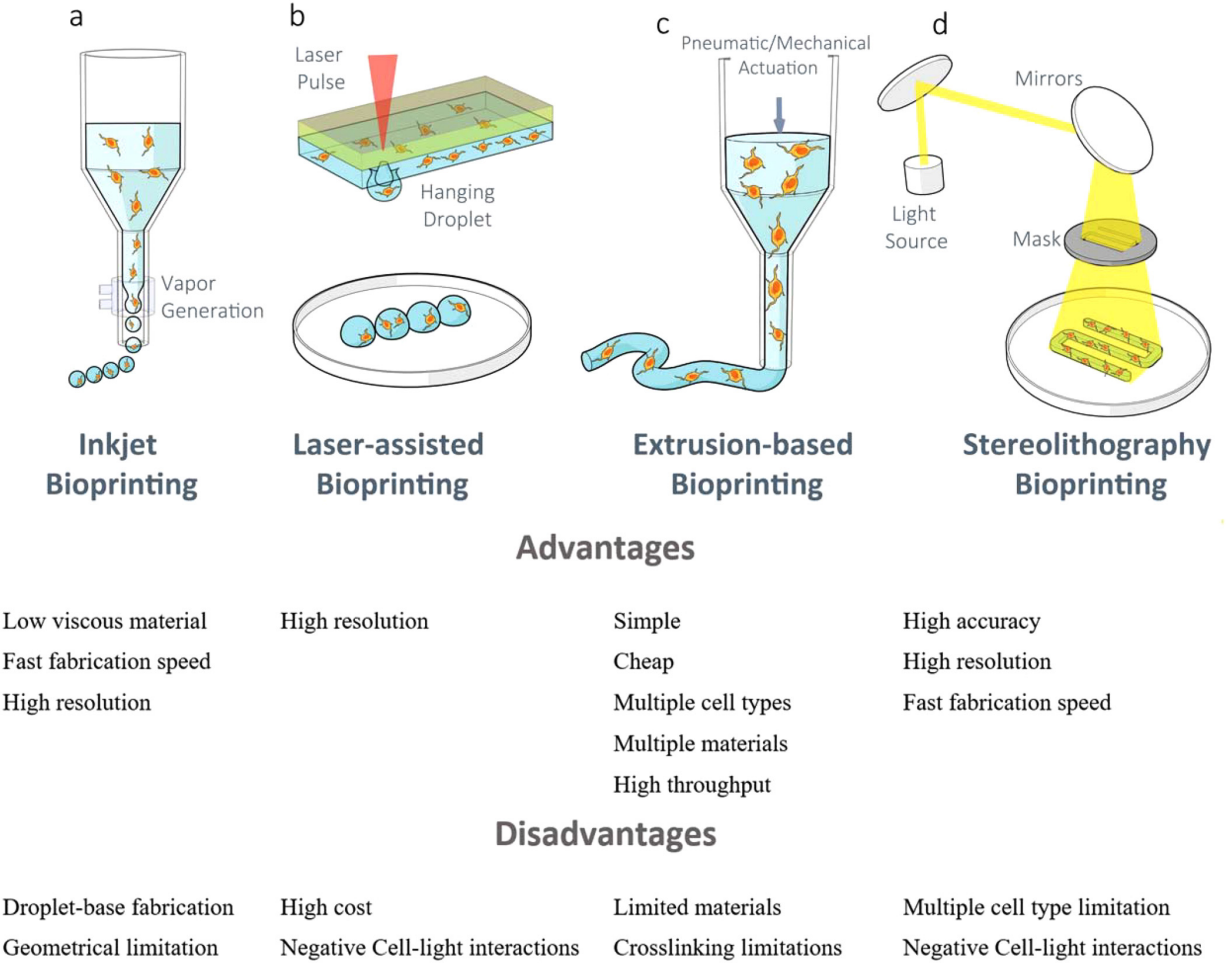
3D bioprinting technologies for neural tissue bioprinting with the advantages and disadvantages of each system.^70–72^

To date, different bioprinting techniques have been developed and used for printing different synthetic tissues and organ models. The major technologies used for bioprinting are inkjet, laser-assisted, extrusion, and stereolithography. Each of these printing techniques has specific strengths and limitations. Figure 3 depicts 3D bioprinting technologies for neural tissues in a nutshell.

### A. Inkjet bioprinting

Inkjet 3D printing, which is very similar to 2D inkjet printing, is the first technique that was utilized for bioprinting.^58^ In this technique, the hydrogel pre-polymer solution either with cells or without cells is stored in the cartilage ink as a bioink source. Then, the cartilage is connected to the printer head. The printer heads are buckled by a piezoelectric or thermal actuator and squeezed to create droplets of different sizes.^45^ A 3D construct is then produced by stacking those droplets on top of each other in a per-defined pattern. The advantages of the inkjet bioprinting method are high cell viability (80%–90%),^59–61^ low cost, and high printing speed. Cell viability is shown to be high as there is less mechanical stress on the cells. Also, printing speed can be enhanced as the printer heads are mostly able to function in a parallel mode. The disadvantages of inkjet bioprinting include the inability of printing high viscous material (>5 MPa/s) and high cell density (1 × 10^6 cells/ml). The high density of cells increases viscosity of bioink and the chance of settling of cells, which results in clogging the printer head.^62–64^

### B. Laser-assisted bioprinting

The main part of the laser-assisted printing system is a donor layer, which is composed of a ribbon structure on the top and a bioink layer suspended at the bottom. During printing, a small area of the absorbing layer is stimulated by a focused laser pulse. This laser pulse evaporates a slice of the donor layer, resulting in a high-pressure bubble at the interface of the bio-ink layer and pushing the suspended bioink. The hanging bioink droplet is received on the substrate and finally crosslinked. One of the major advantages of a laser-assisted printing technique is the high cell viability. The reason is that there is no contact between the dispenser and the bioink. Therefore, there is no mechanical stress on the cells, which also results in increasing the cell density.^45^ Another advantage of this method is the ability to use a high viscous material. In that regard, cells can also be printed more precisely; cells can be manipulated individually and arranged into precise patterns. However, the laser diode intensity and optical setup (e.g., mirrors and lenses) with high precision are expensive when compared with other nozzle-based bioprinting devices.^45^

### C. Extrusion-based bioprinting

The extrusion bioprinting technique is a modified version of inkjet bioprinting. In order to print a high viscous material, extrusion bioprinting uses either a mechanical screw plunger or an air-force drive. The material is printed in a continuous line rather than a droplet-based pattern.^45^ One of the advantages of extrusion bioprinting is the ability to print a wide range of high viscous martials (ranging from 30 to 6 × 10^−7^ mPa s)^8,65^ with high cell density. Extrusion bioprinting is a relatively more simple and cheaper method compared to other bioprinting technologies. Extrusion bioprinting also delivers good compatibility with chemically, thermally, and photo-crosslinkable hydrogels.^66,67^ Also, by simply replacing a custom-designed printhead, the extrusion method can print multi-material constructs, coaxial printing. The main disadvantage of extrusion bioprinting is the low cell viability as the encapsulated cells are exposed to high mechanical stress.^68^

### D. Stereolithography bioprinting

In this technique, a layer of hydrogel is exposed to a light pattern (through masks), which caused the hydrogel in that area to be crosslinked. This process can be repeated through vertical translation of the stage. The key advantage of this technique is the ability to print much higher resolutions in a shorter time as the entire layer is exposed at the same.^45^ It has been shown that the stereolithography bioprinting system can attain 100 *μ*m resolution in less than 1 h while maintaining very high cell viability (90%).^69^ The main disadvantage of this technique is the difficulty in positioning the cells. There is also a limitation in printing multi-material structures.

### E. Integration of microfluidics in bioprinting

Integration of microfluidic systems in the field of neural tissue modeling is not limited solely to 3D model biofabrication. In fact, it opens up a new opportunity in drug delivery and pharmaceutical applications. Microfluidic systems provide cell-level access to *in vitro* neural tissues and provide the ability to manipulate cell–cell interactions in regard to chemical and topological cues. Low volume handling of costly biological samples^73^ as well as the high-level control over the oxygen and nutrition transfer, in addition to real-time monitoring of microenvironment compositions and cells behaviors, are the valuable capabilities of microfluidic systems. Considering the sophisticated system of neural tissues consisting of different cell types, microfluidic devices can be used in Alzheimer's disease (AD) triculture of neurons, astrocytes, and microglia.^74^ The combinatory studies are not necessarily limited to different cell types when it comes to the application of microfluidics. In fact, it can include different biomaterials with complementary properties in multiple layers.^75,76^ The microfluidic systems can even extend to cell-based models, such as brain organoids. Formation of uniform and high-throughput brain organoids derived from human induced pluripotent stem cells (hiPSCs) is no longer a tedious practice with a low rate of failure.^77,78^ In fact, studies on the differentiation of neural stem cells are another emerging area that benefits from the introduction of microfluidic systems. In that regard, engineering a sophisticated vasculature network is one of the important factors affecting cell differentiation.^79^ Microfluidics can also be used as a tool to recreate important features of the native neural ECM, such as concentration gradients. Durotactic gradients can be precisely and quantitatively modeled by varying the crosslinking rate of collagen as the cell scaffold material.^80^

## IV. NEURAL BIOINK

Numerous composite hydrogels are successfully implemented to culture neural cells in 3D structures. There are two main factors regarding the characteristics of the biomaterials (bioinks): printability and gelation ability. For printability, bioink should have shear thinning behavior with a specific viscosity range. Also, bioink must be crosslinkable to form a semisolid gel, which is required to create solid/semi-solid construct out of a liquid ink. In addition to these two factors, the bioink must be suitable for neural tissue culture (neural bioink). In this section, we first focus on the properties of biomaterials used to create 3D neural tissue models. Then, we review a range of printable bioinks that could be used in a form of blend or mixture with neural biomaterials to create neural bioinks. Finally, we cover a few prominent studies that developed neural bioinks and successfully created 3D printed neural tissue models.

### A. Biomaterial for a 3D neural tissue model

Hydrogels are the main type of biomaterials used to create *in vitro* neural constructs. Their tissue-like mechanical/chemical structure (such as high porosity and low stiffness) and their physical/chemical properties make this class of biomaterial suitable for fabricating neural tissue models. Some of the most prominent hydrogels include agarose,^24,81–83^ collagen type I,^84–88^ hyaluronic acid,^89–91^ PEG (polyethylene glycol),^22,89,90^ chitosan,^90,92,93^ alginate,^41,94,95^ silk fibroin,^23,96–99^ and methylcellulose.^100,101^ Most of these hydrogels have the basic characteristics needed for neural cell cultures. That includes low stiffness, the ability to provide sufficient oxygen and nutrition, cell attachment sites (not irregulating neural phenotype),^8^ and waste diffusion.

Mechanical stiffness of the hydrogel can be tuned to match that of the neural tissue, ranging from 1 to 400 kPa.^23,102,103^ Among the above-mentioned natural hydrogels, collagen type I, hyaluronic acid, and chitosan contain endogenous cell attachment sites, such as arginine–glycine–aspartic acid (RGD) and the amino acid sequence.^104–106^ Hydrogels that do not have endogenous cell binding sites are usually mixed with those that do.^107^ Synthetic hydrogels, on the other hand, are mostly used in a modified form in which cell binding proteins are covalently incorporated into the hydrogel chemical structure through available chemical moieties.^106–108^

Several studies have reported the neural cell culture in hydrogels functionalized with laminin and fibronectin.^23,81,82,90,100^ Most of hydrogels can be managed to have a similar protein diffusion rate as compared to the brain tissue. Collagen type I constructs, as an example, have shown to have a protein (bovine serum albumin) diffusion rate of 2–9 × 10^−4^ mm^2^ s^−1^ when it is made with a concentration of 1%–4.5% collagen gel.^109^ In the past, collagen type I was widely used to create *in vitro* brain tissue models.^30,110,111^ This is mainly due to the fact that collagen type I has a higher neural survival ratio and facilitates more neurite outgrowth as compared to other types of ECM.^112^ Collagen type I also has endogenous RGD moieties that facilitate greater neural cell attachment, while it does not require additional cell binding protein (such as laminin). 3D collagen cultures have shown to facilitate the differentiation process of neural stem cells into neurons, astrocytes, and oligodendrocytes.^113^ Similarly, collagen-entrapped progenitors and stem cells can effectively expand and generate neurons that can develop neural polarity, neurotransmitters, ion channels and receptors, and excitability.^114^ These results suggest that collagen is a suitable ECM for guided stem cell differentiation toward favorite glial phenotypes or neurons. In fact, it is an attractive biomaterial to model highly migratory and permissive cell growth environments (such as modeling glioblastoma).^115^ Another example of collagen type I application is to create a 3D endothelial structure to model the blood–brain barrier and vascular compartments of the brain.^110,116^ Collagen, however, has a fast degradation rate and that is the reason why it is not found in the brain tissue. Collagen is also hard to pattern, and therefore, it is not easy to manipulate to create complex structures using conventional fabrication methods.^117^ This can affect the reproducibility of the tissue models.

Another biomaterial that is widely used to create brain tissue models is hyaluronic acid (HA).^89^ HA is an attractive biomaterial for neural models mainly because of its abundant presence in the brain ECM.^7^ Similar to collagen, HA has been used to model glioblastoma. It has been shown that by controlling the hydrogel stiffness, HA can guide differentiation of neuron progenitors toward neuron or glial cells.^118^ As another example of controlled differentiation, fetal and adult progenitors (3D cultured in HA) have shown tendency to differentiate into glial cells and neurons, respectively.^119^ HA has also been shown to preserve neural stem cell quiescence and discourage differentiation (unlike progenitors).^120^ HA, however, is shown to be successful for long cultures mainly because it inhibits the neurite outgrowth tendency.^121^ Overall, HA is an attractive biomaterial that can be used with other biomaterials in a composite form to create complex neural tissue models.

Agarose and PEG are bioinert hydrogels with tunable stiffness. ECM-derived peptides or growth factors are used to functionalize the hydrogels through covalent coupling of bioactive molecules. Therefore, they can be used to observe individual effects of different parameters on the fate of cells.^22,81,82,118,122^ These two polymers, however, cannot be modified by cells unless they are altered with degradable peptide moieties or crosslinked with hydrolysis-prone chemistry.^123^

Silk protein has been popular for neural tissue applications because of its functionalization capability, biocompatibility, and tunable stiffness.^30,124,125^ However, silk bioink does not meet the requirement of bioprinting since shear thinning behavior of silk takes place at low concentrations where it is not enough for printing (low viscosity).

### B. Printable biomaterial

There is an extensive range of biomaterials that has been used for bioprinting. Currently, materials used for bioprinting are mainly alginate, poly(ethylene glycol) diacrylate (PEGDA), hyaluronic acid, chitosan, fibrin, silk fibrin, gelatin, agarose, methylcellulose, and collagen. Biomaterials should have two major characteristics to be considered for printing: printability and gelation. Gelation might not be compatible with every printing technology. Also, every biomaterial may require different conditions to go through the gelation process, and hence, they can only be used with their compatible printing setup to create a 3D printed construct. In this subsection, a few prominent gelation mechanisms used in 3D bioprinting are covered.

Gelation of an ink in 3D printing preserves the shape of the printed structure and facilitates required structural supports to develop a 3D construct in bioprinting. There are three types of gelation: physical, chemical, and enzymatic gelation. Physical gelation is based on reversible interaction, while chemical and enzymatic gelation is based on the formation of a covalent chemical bond. Physical gelation happens by a temperature change, a PH change, or ionic crosslinking, while chemical gelation happens by chemical reactions and formation of chemical bonds (e.g., photocrosslinking). Table I lists a variety of hydrogel materials (e.g., alginate, gelatin, GelMa, collagen, fibrin, Matrigel, etc.) used in bioprinting with different techniques, including extrusion, inkjet, and laser-induced printing. These materials are classified based on the crosslinking method.

**TABLE I.** List of hydrogel materials used in bioprinting (classified based on crosslinking methods).

Hydrogel	Fabrication method	Gelation method
Alginate^63,133,134^	Laser-induced forward transfer	Ionic
Alginate^135,136^	Inkjet	Ionic
Alginate/collagen type 1^137^	Inkjet	Ionic
Collagen type 1^138^	Inkjet	Thermal
Fibrinogen/ collagen type 1^139^	Inkjet	Enzamatyc
Poly(ethylene glycol) dimethacrylate^59^	Inkjet	Photo
Agar^140,141^	Extrusion	Thermal
Agarose^142–145^	Extrusion	Thermal
Alginate^146–148^	Extrusion	Ionic
Alginate/fibrin^141^	Extrusion	Ionic/enzymatic
Alginate/gelatin^149^	Extrusion	Thermal/ionic/chemical
Collagen type 1^150,151^	Extrusion	Thermal
Collagen type1^152^	Extrusion	pH (sodium bicarbonate)
Gelatin^152^	Extrusion	Thermal
Gelatin methacrylamide^153^	Extrusion	Thermal/photo
Gelatin/alginate^154^	Extrusion	Thermal/ionic
Gelatin/alginate/fibrinogen^155^	Extrusion	Thermal/enzymatic/ionic/chemical
Gelatin/alginate/fibrinogen^156^	Extrusion	Thermal/ionic/enzymatic
Gelatin/chitosan^157^	Extrusion	Ionic/chemical
Gelatin/chitosan^158^	Extrusion	Thermal
Gelatin/fibrinogen^159^	Extrusion	Enzymatic
Gelatin methacrylamide/hyaluronic acid^153^	Extrusion	Thermal/photo
Gelatin methacrylamide/gellan^160^	Extrusion	Ionic/thermal/photo
Matrigel^161^	Extrusion	Thermal
N-isopropylamide and polyethylene glycol^162^	Extrusion	Thermal
Poly(ethylene glycol) diacrylate^143^	Extrusion	Photo

#### 1. Physical crosslinking

Physical crosslinking can be defined as an entanglement of high molecular polymer chains, hydrophobic interactions, or hydrogen bridges.^126^ Some of the most common physically crosslinked hydrogels are agarose, alginate, chitosan, collagen, gelatin, Matrigel, and Pluronic (F-127). Among different crosslinking methods, physical crosslinking has received more attention in tissue engineering due to the fact that the physical crosslinking polymers are crosslinked without the use of any exogenous agents. Therefore, chemical toxicity is decreased in such polymeric hydrogels. Therefore, they can provide a more friendly environment for embedded cells, proteins, and other biologics.^127^

#### 2. Chemical crosslinking

The process of chemical crosslinking of hydrogels involves formation of covalent bonds between chains. Chemically crosslinked hydrogels have better mechanical stability than that of the physically crosslinked hydrogels. An external crosslinking agent is used in the chemical crosslinking of hydrogels to induce the gelation reaction. However, this may result in inducing an undesirable reaction with the hydrogel surface or cytotoxicity.^127^ One of the main mechanisms for chemical crosslinking is photo-crosslinking.^128^ Methacrylated gelatin (GelMA) and poly(ethylene glycol) diacrylate (PEGDA) are two types of commonly used polymers capable of photo-polymerization.

#### 3. Enzymatic crosslinking

The process of protein crosslinking comprises chemical, enzymatic, or chemoenzymatic formation of new covalent bonds between polypeptides.^129^ *In vivo* covalent modifications of proteins are mostly catalyzed by specific enzymes that have evolved to perform their specific tasks.^129^ Fibrin is the most common enzymatically crosslinked hydrogel in tissue where it is composed of fibrinogen and thrombin (the major precursors of blood clotting) engineering.^130,131^ In essence, thrombin is a protease, and Ca^2+^ is a dependent enzyme that converts fibrinogen into fibrin. Also, the cell adhesive and biocompatibility properties of fibrin have made it a widely used bioink in bioprinting.^132^

### C. Neural bioink

Neural tissue bioprinting methods can be categorized into two groups: two-step printing and advanced printing. Previously, two-step printing techniques have been used to fabricate neural tissues.^163^ In such a technique, scaffolds are first printed and then a layer of cells is seeded within the construct. Recently, advanced printing techniques are used to print neural tissues in one step. In these methods, cells that contained bioinks are printed to create more complex structures of neural tissues with spatial organization of cells within the construct. In Sec. IV C 1, we covered neural tissue models that are fabricated using two steps of printing and advanced printing methods.

#### 1. 3D printed neural tissue using two-step printing

Low-level light therapy (LLLT) can be integrated with 3D printing to fabricate neural tissue models and repair neural degeneration.^163^ The scaffold composed of gelatin methacrylate (GelMA) and polyethylene (glycol) diacrylate (PEGDA) is first printed using a stereolithography-based 3D printer. Then, neural stem cells (NSCs) are seeded on the 3D-printed scaffold followed by applying a red laser light. The cell response to the red light can be studied by detecting cell proliferation and intracellular ROS synthesis. In addition, the differentiation of NSCs can also be examined by immunocytochemistry and a real-time quantitative reverse transcription polymerase chain reaction (qPCR). It is important to realize the dose dependence of the NSC response after laser exposure. In essence, it is shown that 15 s stimulation promotes cell proliferation, while LLLT is suppressive for higher amounts (such as 60 and 90 s). In addition to the proliferation effect, light stimulation promotes NSC neuronal differentiation and inhibits generation of glial cells as illustrated by the immunocytochemistry study and gene expression testing. Overall, the low-level light stimulation increases cell proliferation and expression of neural specific marker TUJ1.^163^

**FIG. 4.**
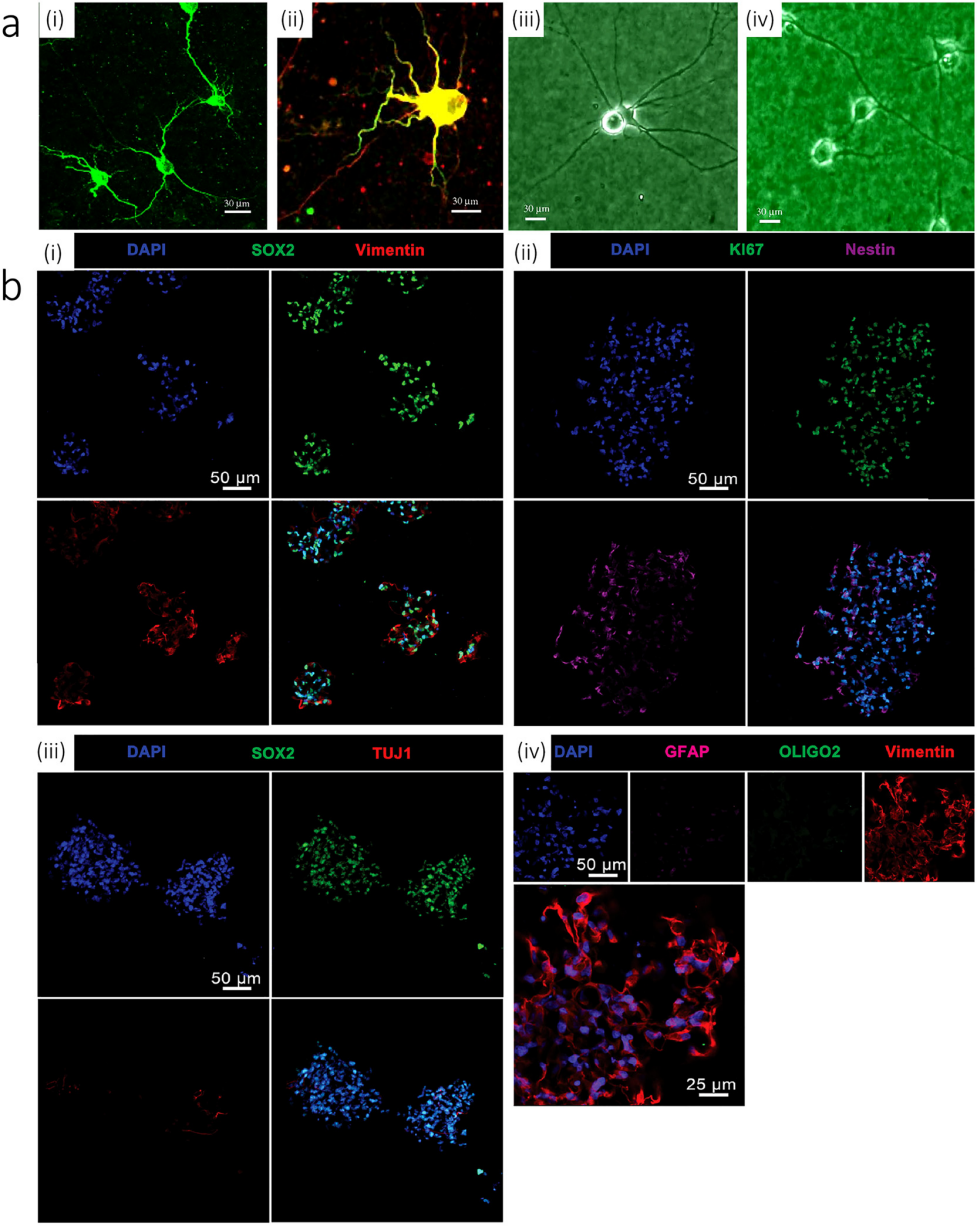
[a (i)] The cell bodies and dendrites of rat embryonic cortical neurons were immunoreacted with MAP2 monoclonal antibodies (green). [a (ii)] The cell bodies and dendrites of rat embryonichippocampal neurons were immunoreacted with anti-MAP2 monoclonal antibodies (green), while the axons of the neurons were immunoreacted with anti-neurofilament monoclonal antibodies (red). Morphologies of printed hippocampal neurons [a (iii)] after 13 days of culture and printed cortical neurons [a (iv)] after 9 days of culture, both recorded by phase contrast imaging. Reproduced with permission from Xu *et al*., Biomaterials **27**, 3580 (2006). Copyright 2006 Elsevier.^164^ (b) Immunophenotyping of hNSCs encapsulated within an optimal gel construct 3 weeks after printing. [b (i)] hNSCs stained with DAPI colocalized with SOX2 and expressed vimentin. [b (ii)] Cells also expressed nuclear proliferation marker KI67 and hNSC marker nestin. [b (iii)] hNSCs expressed negligible levels of differentiated neuron marker TUJ1. [b (iv)] hNSCs expressed negligible levels of differentiated astrocyte and oligodendroglial lineage markers GFAP and OLIGO2, respectively. Reproduced with permission from Gu *et al*., Adv. Healthc. Mater. **5**, 1429–1438 (2016). Copyright 2016 John Wiley & Sons.^6^

Inkjet printing can be utilized to directly print cells on the biopaper layer.^164^ It is shown that 74.2 ± 6.3% cell viability can be achieved after 8 days. Also, expression of neural marker MAP2 can happen after 15 days. Within this time span, neurons develop voltage-gated sodium and potassium channels. Additionally, a 3D neural sheet can be fabricated by alternately printing fibrin gels and NT2 cells. In that regard, a layer of fibrinogen followed by thrombin on top for polymerization is printed. A single layer of a NT2 neuron cell is inkjet printed with a specific pattern on top of the hydrogel layer. If the entire process of alternate printing of thrombin and NT2 neurons is repeated for five cycles, it results in a 3D neural sheet [neurite outgrowth after 12 days is depicted in Fig. 4(a)].^164^

A digital micromirror-array device (DMD) printing technique is another method to fabricate engineered structures mimicking the architecture of the native nerve.^165^ Native extracellular components, such as hyaluronic acid (HA), can be used for fabrication of scaffold. HA can be modified by the methacrylate group to achieve photo-crosslinkable material. Glycidyl methacrylate modified hyaluronic acid (GMHA) can be implemented to fabricate the tissue scaffold. Then, the fabricated scaffold is modified to become cell adhesive since HA inherently is not cell adhesive. Next, adhesive proteins, such as laminin, are grafted on the surface of the scaffold. Finally, Schwann cells are seeded on the scaffolds. The adhered cells are shown to maintain viability after 36 h. It is also reported that this method could be used to make fluorescent microparticles as a model for the growth factor gradients, which guides developing neurites.

#### 2. 3D printed neural tissue using an advanced 3D bioprinting method

In this method, material and cells are printed simultaneously to create heterogeneous tissue models. These models greatly benefit high-throughput clinical applications and drug testing. To create a 3D neural tissue using human neural stem cells (hNSCs), as an example, an extrusion printing technique with polyacrylate-based bioink may be used.^6^ In that regard, the bioink is comprised of alginate, carboxymethyl-chitosan (CMC), and agarose in which alginate and agarose are used as structural support. The agarose is mainly used to confer bioink suitable viscosity during printing and prior to gelation, whereas alginate is used to enable gelation after printing by immersing the printed construct in a cation bath (e.g., the calcium chloride solution). CMC, which is a water soluble derivative of chitosan, is utilized in the bioink to ensure cell survival. It is shown that hNSCs have good viability and continue to renew and proliferate within the printed construct for 10 days. After the initial 10 days of culture, hNSCs are differentiated into glial cells and neurons for two weeks. The pore size of bioink [alginate (Al), carboxymethyl-chaisson (CMC), and agarose (Ag)] is another critical factor. It is stated that different concentrations of carboxymethyl-chaitason (CMC) can cause variable porosity: 5% and 3.5% (w/v) CMC results in highly and lightly porous surfaces, respectively. 2% or less (w/v) CMC gels causes negligible or no pores. The compressive Young's modulus of the optimized ink (5% w/v CMC, 5% w/v Al, and 1.5% w/v Ag) is 7.5 kPa. For the mentioned system, the seeding density can be around 5 M/ml with the maximum printing resolution of 500 *μ*m. The printed hNSCs are differentiated into neurons and neuroglia after 10 days post printing. A Tuj1 marker should be used to label neuron cells. Also, GABA and GAD (glutamic acid decarboxylase) markers are used to label GABAergic neurons. In addition, for staining neuroglia cells, OLIGO2 and GFAP are implemented [see Fig. 4(b)].^6^

**FIG. 5.**
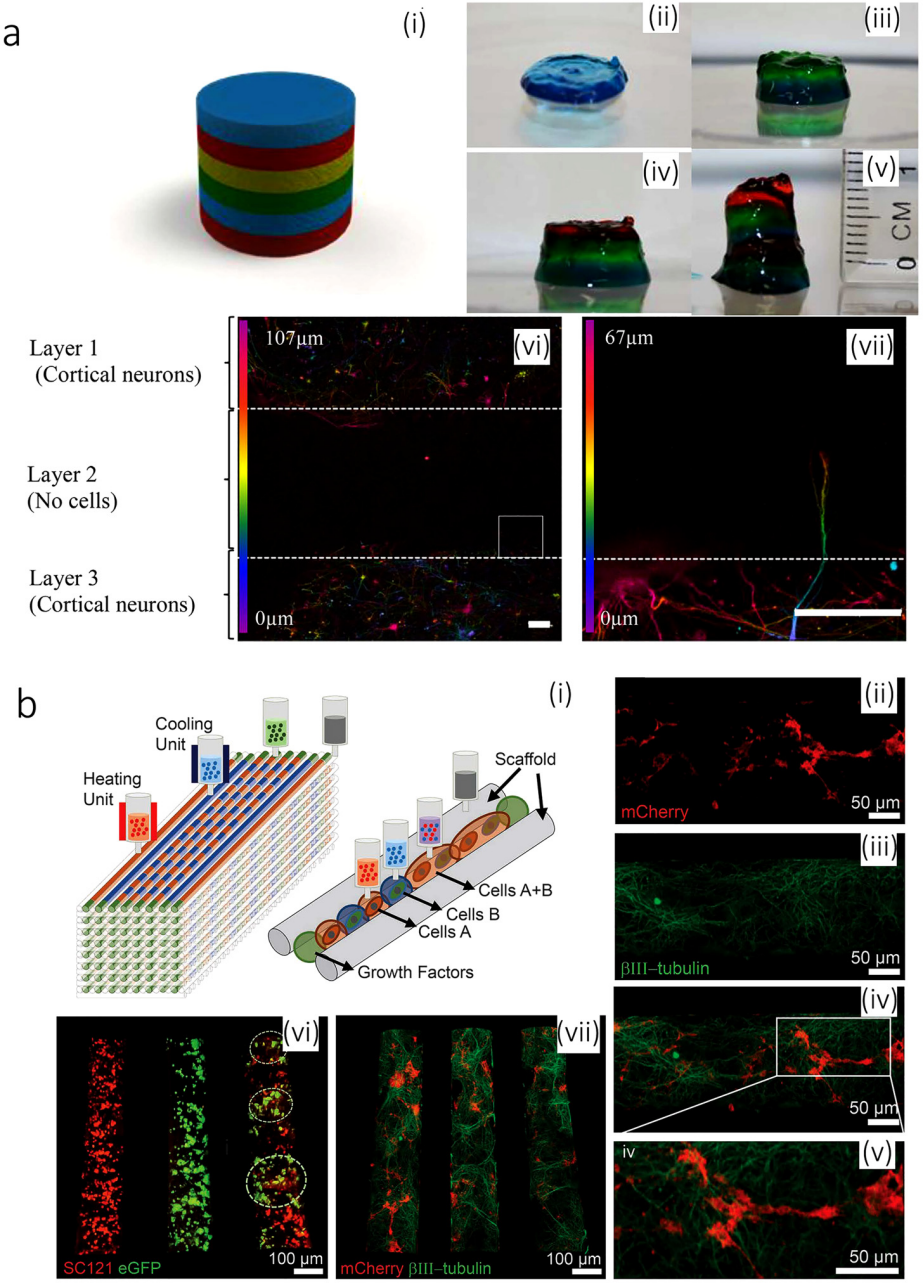
(a) Printed brain-like layered structures. [a (i)] Illustration of the proposed brain-like layer structure. [(a) (ii)]–[(a) (vii)] The printing process to create a brain-like structure. Each color represents a layer. [(a) (vi)] Confocal microscope images of neurons in different layers after 5 days of culture. The image is colored for the distribution of the cells through the *z*-axis in the bioink RGD-GG gel as indicated. [(a) (vii)] The expanded view of the area from the square, showing an axon penetrating into the adjacent layer. Scale bars represent 100 mm. Reproduced with permission from Lozano *et al*., Biomaterials **67**, 264–273 (2015). Copyright 2015 Elsevier.^166^ (b) 3D bioprinted iPSC-derived neuronal and glial progenitor cells after *in vitro* culture. [(b) (ii)–(b) (iv)] The image of 3D printed sNPCs and OPCs in a channel after 7 days of culture showing axon projections in close proximity to the OPCs. OPCs express mCherry (red), sNPCs differentiate into neurons and express the β3III-tubulin (green), and the merged image is shown along with a close-up. [(b) (vi)] Distribution of cell types in specific channels: sNPCs only (left), OPCs only (middle), and sNPCs and OPCs (right). sNPCs are detected with human-specific antibody SC121 (red), and OPCs express eGFP (green). The image was taken 24 h post printing. [(b) (vii)] sNPCs and OPCs co-printed in a scaffold after 4 days of culture. β3III-tubulin shows axonal projections down the channels, and the OPCs express mCherry. Reproduced with permission from Joung *et al*., Adv. Funct. Mater. **28**, 1801850 (2018). Copyright 2018 John Wiley & Sons.^167^

Neural bioinks can be comprised of only 0.5% gellan gum [modified with arginine–glycine–aspartic acid (RGD)] in water.^166^ The gel is crosslinked once exposed to a calcium chloride solution. In such a system, the cell viability of 75% can be reached for a multi-layered neural model, consisting of discrete layers of primary cortical neural cells encapsulated in a hydrogel. The printing device can be as handy as a hand-held printer with a coaxial nozzle. The printing cell density can reach up to 1 M/ml. Despite the promising results reported for this strategy, cell viability and staining after 5 days are not provided [see Fig. 5(a)].^166^

3D neural construct can be printed by serially depositing the scaffold ink and multiple cell-laden bioinks in a layer-by-layer manner. With a 3D silicone scaffold as the base, a cell-laden Matrigel is printed to create multiple channels for mimicking the parallel fibers similar to those in the spinal cord. Two neural lineage stem cells [iPSC-derived spinal neuronal progenitor cells (sNPCs) and oligodendrocyte-progenitor cells (OPCs)] with an alternative dot arrangement are investigated for this method. For such a system, different hydrogel materials, such as GelMA, gelatin/fibrin, and Matrigel, are characterized. It is shown that GelMA and gelatin/fibrin are good for fibroblast but not for neurons. However, Matrigel is a good choice for neurons [see Fig. 5(b)]. The results of the calcium imaging show the interconnectivity of neuron cells in their structure. Printing sNPCs and OPCs onto the scaffold and culturing for 7 days, the outgrowth of axons with the attendance of OPCs can be observed within the printed microchannels using the NeuN neuronal mature marker. These results indicate the differentiation of the printed sNPCs into neurons with extended axons. Finally, to test the activity of these neuronal networks, physiological spontaneous calcium flux studies can be performed [see Fig. 5(b)].^167^

**FIG. 6.**
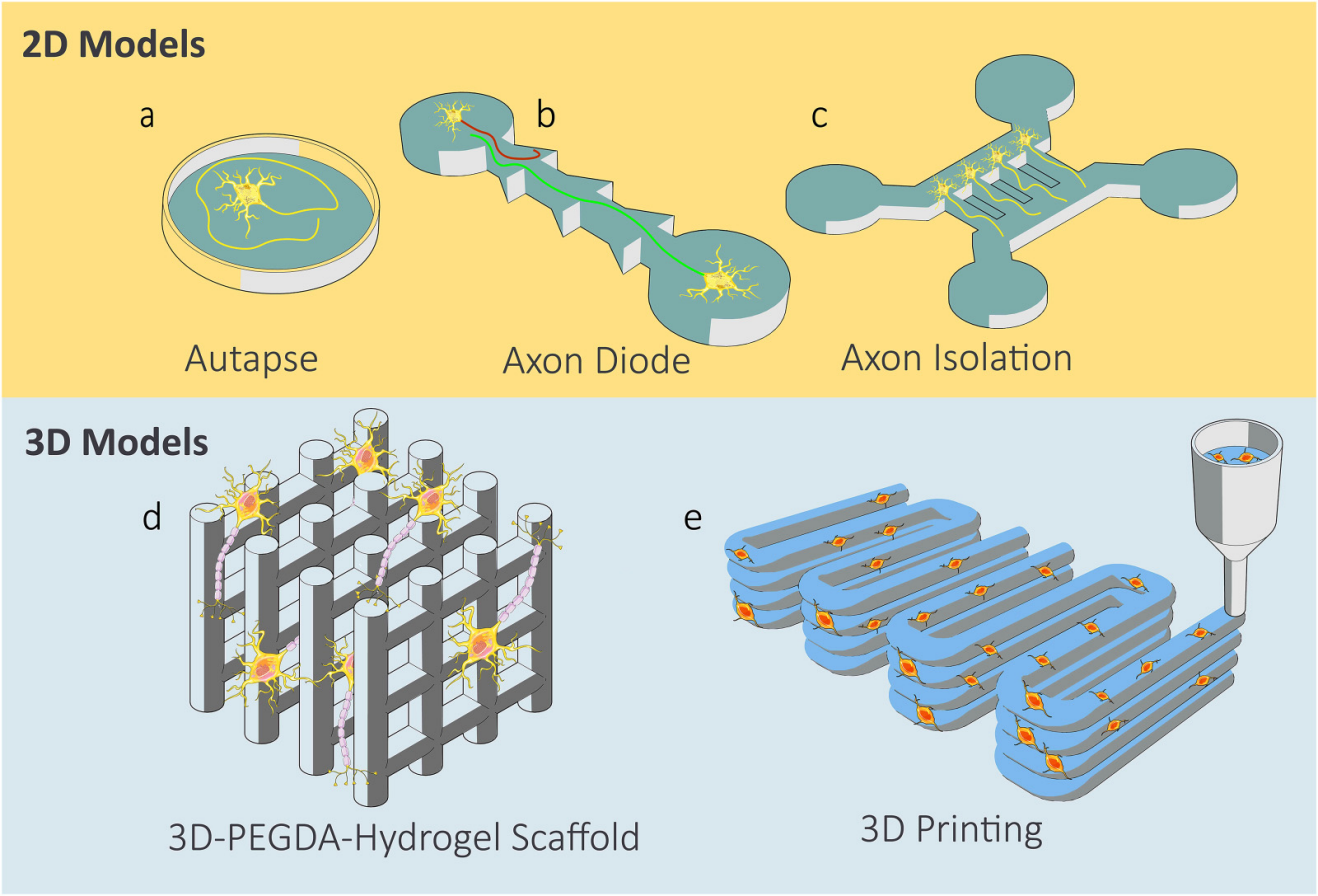
2D on-chip and 3D neural tissue models.^79,168^ (a) Self-synapse. (b) Axion diode model and the unidirectional neuron connections. (c) Axon–soma isolation. (d) Prefabricated stereolithography scaffold and growth of neurons in the pores. (e) 3D printing of a cell contained hydrogel.

After reviewing the neural bioink, a general representation of the most on-chip and 3D models in neural tissue engineering is provided in Fig. 6. Neural tissue engineering is an emerging field with huge potential in bioprinting and *in vitro* model developments. Regardless of the application of the *in vitro* neural models, whether it is an on-chip disease model or a 3D block with implantation and pharmaceutical applications, they can be seen in either of the forms represented in Fig. 6.

## V. FUNCTIONALITY OF A 3D PRINTED BRAIN MODEL

Engineered 3D brain models should be evaluated with appropriate techniques after enough maturation of the neural stem cells. The functionality of these models should be determined by the maturity of the neural cells using antibodies for each cell-specific marker molecule or neuronal activities. The neuronal activities are elicited by the oscillation of the ion dynamics between inside and outside of the cell membrane. The challenging point is that the 3D constructs are thicker than the conventional 2D monolayer cultures. Therefore, researchers cannot readily apply the same methods to 3D-engineered tissues. In essence, the thickness of an artificial tissue might obstruct our observations using standard optical methods or even measuring action potential of the cell surface. It is good to note that when measuring action potential, the electrodes should directly contact the cell surface. Considering the fact that most cells are located at different *z*-positions, the standard 2D multi-electrode array cannot be used either. Crosslinked bioinks represented by GelMA are nebulous so that both fluorescent excitation and emission light are inhibited from passing though the constructs. Besides, the antibodies for biomarkers cannot easily access the molecules deep inside the hydrogel. Therefore, the reaction time with antibody should be increased more than the standard immunostaining techniques. Subsection V A lists the appropriate tools used to evaluate the cell conditions inside the hydrogel.

### A. Cytotoxicity/proliferation assay and live cell imaging

Regarding the thickness of the artificial 3D brain models, nutrients and oxygen might not be able to access the deep-seated cells. Besides, bioinks should be crosslinked by UV irradiation or a photo-initiator, which might decrease the viability of the cells. Thus, cell viability in 3D brain models should be evaluated with the highest priority. MTT or related tetrazolium salts (XTT, MTS, WSTs) are frequently used for estimating the living cell population. Living cells reduce the tetrazolium salts and converted them into formazan dyes. Generated formazans are quantified with absorbance of visible light. Resazurin-based reagents, represented by PrestoBlue and Alamar Blue, have also been used for quantifying the living cell numbers. Those chemicals are reduced by living cells, and red fluorescent resorufins are generated. Both tetrazolium- and resazurin-based chemicals can be used in the same way, i.e., add into the culture medium/buffer, let cells reduce the parent chemicals, and release the reduced dye to the extracellular space. The main advantage of these chemicals is the easiness to use and quantify the entire 3D construct. The disadvantages of these chemicals are (i) the slow rate of the reduction reaction in the 3D models (as this rate depends on the speed of both parent chemicals and reduced dyes through the biomaterials) and (ii) their inability for dead cell evaluation. Therefore, these chemicals can only be used to evaluate the relative toxicity or proliferation ability among different 3D hydrogels or additives. Calcein-AM and ethidium homodimers, on the other hand, can be used for evaluation of both live and dead cells. Essentially, non-fluorescent calcein-AM can easily enter the living cells, and the intracellular esterase hydrolyzes the calcein-AM to intensely green fluorescent calcein. Ethidium homodimers cannot enter living cells, but it can enter damaged cells and elicits strong red fluorescence upon binding DNA. By combining these two chemicals, both living and dead cells can be evaluated simultaneously using confocal microscope and cell counting tools from 3D stacked image sequences (e.g., ImageJ plugin 3D Objects Counter).^169^ Neural progenitor cells or neuron-specific live cell imaging reagents are now commercially available (CDr3 or NeuO, respectively). CDr3 is supposed to bind to fatty acid binding protein 7 (FABP7), which is expressed in neural progenitor cells. The mechanism of binding to neurons is not known, but NeuO can selectively bind to neurons. Using these chemical and confocal microscopes, researchers can depict the living 3D neuronal network.

### B. Immunostaining

Staining of neural biomarkers is an informative tool to determine the type and the state of the cells in the model and to evaluate the accuracy of the model in representing certain functions of the part in CNS. They can also be used to track the development, maturity, and functionality of neural cells in the bioprinted construct. Here, we cover the most common types of biomarkers used in brain models (Table II).

**TABLE II.** List biomarkers used in brain models.

Biomarker	Marker of
Nestin^170^	Neural stem cell and progenitor
Class III β-tubulin and its antibody TUJ^171^	Premature or differentiated neural cells
Microtubule-associated protein 2 (MAP-2)^172^	Mature neuron
Gamma aminobutyric acid (GABA) and its enzymes GAD65/67^174^	Mature interneuron
Tyrosine hydroxylase (TH)^175^	Mature dopaminergic neurons
Tryptophan hydroxylase (TPH)^176^	Serotonergic neurons
Choline acetyltransferase (ChAT)^177^	Differentiated cholinergic mature neurons
Glial fibrillary acidic protein (GFAP)^178^	Cell death or astroglia injury
Oligodendrocyte transcription (OLIG1 and OLIG2)^179^	Mature oligodendrocytes
CD11b, CD45^180^	Microglia cell
CD86, inducible nitric oxide synthase (iNOS)^180^	M1 microglia cell
Arginase 1 (ARG1), CD206^180^	M2 microglia

#### 1. Neural progenitor cell marker

Nestin is a neural stem cell and progenitor cell marker, which is an intermediate filament protein in the cytoskeleton. It is expressed in the undifferentiated CNS during development as well as normal and adult CNS and in CNS tumor cells. Additionally, it is known that nestin is expressed in proliferating endothelial progenitor cells (EPCs) and is not expressed in mature endothelial cells.^170^ Therefore, when a vascularized brain model is made, careful counterstaining of endothelial cells might be needed to certify the existence of the neural stem cells in the constructs.

#### 2. General neuron marker

Class III beta-tubulin is a neuron-specific biomarker that is in microtubules and used to recognize premature or differentiated neural cells. Neuron-specific class III beta-tubulin and its antibody TUJ have been used as a neuron-specific marker but classified as an immature neuron.^171^ A more mature neuron biomarker is microtubule-associated protein 2 (MAP-2), which is a cytoskeletal protein mainly found in soma.^172^

#### 3. Glutamatergic neuron marker

Glutamatergic neurons have an enzyme to produce glutamate in the cells (glutaminase) and a transporter to condensate it in the vacuole in the nerve ending (vGLUT1 and vGLUT2).^173^ They also have the glutamate-gated ion channels, which produce calcium transient upon glutamate binding (NMDAR1 and NMDA2B).

#### 4. GABAergic neuron marker

It is a neurotransmitter released by a GABAeregic interneuron (inhibitor neurons).^174^ GABA is considered a mature neuron marker for GABAregic interneurons. GAD65/67 are two enzymes involved in the production of GABA. They are also considered mature neuron biomarkers.

#### 5. Dopaminergic neuron marker

Tyrosine hydroxylase (TH) is a mature neural biomarker, which is an enzyme involved in the production of dopamine and norepinephrine (NE).^175^ TH is generally used as a marker for dopaminergic neurons.

#### 6. Serotonergic neuron marker

Tryptophan hydroxylase (TPH) is a rate-limiting enzyme for serotonin production in serotonergic neurons.^176^ In essence, serotonin molecules are stored in vesicles, and they are released to synaptic cleft after cells become excited. Those molecules are reuptaken by serotonergic neurons themselves through a serotonin transporter (SLC6A4/5-HTTLPR).^176^

#### 7. Cholinergic neuron marker

Choline acetyltransferase (ChAT) is another mature neural biomarker.^177^ ChAT is an enzyme involved in the production of the neurotransmitter acetylcholine and used as a marker for differentiated cholinergic neurons.^177^

#### 8. Astrocyte marker

In a differentiated neuronal culture, GFAP is only found in astrocytes.^178^ In a native tissue, GFAP is also expressed by ependymal cells during development.

#### 9. Oligodendrocyte marker

Oligodendrocyte transcription factors 1 and 2 (OLIG1 and OLIG2) are known as the specific basic helix–loop–helix factors for oligodendrocytogenesis.^179^ Myelin oligodendrocyte glycoprotein (MOG) is also identified as mature oligodendrocytes.

#### 10. Microglia marker

Microglia serve as immunocompetent cells in the brain.^180^ Cell surface markers CD11b and CD45 are well known steady-state microglia markers. Microglia change their class from a steady state in response to the external stimuli: proinflammatory phenotype M1 or neuroprotective M2 microglia. M1 microglia express CD86 and inducible nitric oxide synthase (iNOS), whereas M2 microglia express arginase 1 (ARG1) and CD206.

### C. Calcium imaging

Calcium imaging is a technique that is used to show the status of calcium (Ca^2+^) of individual cells, tissue, or medium.^181^ This technique has also been utilized for studying the calcium signaling in neural activities in neuron and glia cells. Calcium ions produce versatile intercellular signals that control key functions in all types of neurons.^181^ As an example, the calcium dynamics at presynaptic terminals are accessible to calcium imaging.^182,183^ For this purpose, presynaptic terminals are loaded with an appropriate calcium indicator dye (i.e., fluorescent molecules responding to binding the calcium ions by changing their fluorescent properties). Two main classes of calcium ions are chemical indicators and genetically encoded calcium indicators (GECIs).^184^ Chemical indicators are small molecules that can chelate calcium ions. This group of indicators includes indo-1, fura-2, calcium green-1, fluo-3, and fluo-4. Binding of a Ca^2+^ ion to a fluorescent indicator molecule results in either an emission/excitation wavelength shift or an increase in quantum yield of fluorescence.^181^ Genetically encoded calcium indicators, on the other hand, are fluorescent proteins derived from green fluorescent protein (GFP) or its variants [e.g., circularly permuted GFP, yellow fluorescent protein (YFP), and cyan fluorescent protein (CFP)].^181^ They do not need to be encumbered onto cells; instead, the genes encoding for these proteins can be easily transfected to cell lines. It is also possible to make transgenic animals expressing the dye in all cells or selectively in some specific cellular subtypes. GECIs have been used in the studies of neurons.^185^ Imaging for both types of indicators is very similar: fluorescence microscope (confocal or two-photon microscope) coupled with a high-sensitivity camera is usually used to capture the real-time images of intracellular calcium ion concentrations/oscillations of individual cells. When information in the vertical direction is not needed, the combination of a standard fluorescent microscope and a light source is enough. However, if the specific layer or whole construct is needed to be monitored with high-time resolution, an updated confocal or a two-photon microscope is needed. General confocal microscopy is time consuming for acquiring a single image (scanning one slice at a time in the vertical direction with covering the entire surface in each slice using a conventional galvanometer scanner system). A high-speed resonant scanning system enables much higher time resolution of each vertical slice, imaging with dozens of frames/s. However, volumetric calcium imaging for thick 3D samples is still challenging. Recently, a spinning disk confocal microscopy system has been introduced for high-speed imaging of thick samples. It consists of two different disks with a series of micro-lenses and corresponding pinholes so that around 1000 laser beams can scan each slice simultaneously and finish the process in a millisecond [>1000 frame/s (fps)]. Using this system, rapid calcium ion oscillations can be monitored in dozens of fps, but the optical limitation is still standing on our way: a confocal system can practically observe only up to 200 *μ*m thickness of a sample.

### D. Electrophysiology

Brain activities should be consolidated into the electrodynamics of neuronal networks. When the stimuli input to the postsynaptic potential exceeds the threshold, it causes an action potential in a pulse manner with a very short duration time (around 1–2 ms). An electrode detection system like a patch clump system is a gold standard method for detecting neuroelectric activities, and it can efficiently detect kHz level of oscillations of neuronal membrane potentials. However, it needs special facilities, such as microscope, micromanipulator, and electrical detection system. This method should be operated by the experienced experimenters and basically applied to the single cell except using a patch clump array. A patch clump array is used for the suspended cells. Therefore, it cannot be applied to the 3D brain models easily. Thus, a patch clump system does not suit for the analysis of the artificial brain sample with large thicknesses since it cannot directly detect the neuronal activities of multiple neurons. On the other hand, the methods used for *in vivo* or *ex vivo* brain analysis can be applied to the 3D artificial brain tissue without huge modifications. A Utah array was developed by Normann *et al.*^186^ This was composed of 100 tapered metal tip needles (10 × 10 array of silicon needles with 1.5 mm long and 4.2 × 4.2 mm^2^ interval). It could detect the multi-point neuronal activities of brain while it was indwelled in intracortical space. Although this type of system has the disadvantage of the invasiveness to the brain when it is used clinically, it can be used in basic 3D brain model research for detecting the neuronal activities inside the construct directly. A 2D multielectrode array (MEA) has also been used for detecting the neuronal activities of multiple neurons in a brain slice.^187^ Although it is difficult to detect the neuronal activities of neurons deep inside the 3D brain model, local field potentials (LFPs), which are generated by the group of neurons near the electrode, can be detected by MEA systems. MEA systems are roughly divided into two groups: one is composed of 60–64 electrodes of array on an electrical circuit with 150 *μ*m pitch and the other is a complementary metal–oxide–semiconductor (CMOS)-based MEA system with 10–20 *μ*m pitch. The former systems detect neuronal activities from multiple cells with a single electrode. Sometimes, it can miss the detection of the neuronal activities between the two electrodes due to the less spatial resolution than CMOS MEA systems. CMOS-based MEA systems have tens of thousands of electrodes, but recording electrodes should be configured up to several thousands. The advantage of using CMOS-based MEA systems is that the experimenter can flexibly select the electrodes in accordance with the neuronal activities with high special resolution. Therefore, the information about the subcellular membrane potential can be recorded. Also, the functional neuronal network linkage is visualized as seen in microscopy. Both systems can theoretically give active stimulation to the neurons from the electrodes. Thus, these functions can also be used for the basic research of the brain–machine interface from the external server. Another advantage of using electrode systems is that they detect the action potentials of neurons without labeling, which sometimes confers toxicity to cells or perturbs cell signaling.^188^ Therefore, the electrode system suits for long term analysis of neurons on the sensor surface. The maturation of neurons can be monitored without fixation.

Instead of using an electrode system, membrane potential can be detected by the optical systems, such as a fluorescent microscope. Membrane potential indicator dyes are classified into two different types: fast or slow response dyes.^189^ Fast response dyes can detect the change of the membrane potential in a sub-millisecond manner. However, the change of fluorescent intensities of fast responsive dyes is only 10% per 100 mV. Therefore, the signal to noise ratio is basically very small. On the other hand, the intensity change of slow responsive dyes is much higher than fast responsive dyes up to 1% per 1 mV. However, the change is elicited by the translocation of the dyes beyond the cell membrane upon the membrane potential shift. Therefore, the change of the fluorescent intensity of slow response dyes appears dozens of milliseconds behind membrane potential shift. Required specification of the optical detection system should be almost equivalent to that used in calcium imaging described above. However, higher sensitive camera and high-speed image scanning systems would be needed especially for fast responsive dyes when 3D brain models are evaluated.

## VI. FUTURE OUTLOOK

Bioprinting could enhance the applicability of neural tissues due to its high-throughput process, which can create physiologically precise brain constructs for applications in drug screening and cell therapy. To enhance the field of bioprinting of neural tissues, new neural bioinks need to be developed. Current neural bioinks do not show noble compatibility with neural cells. Collagen, Matrigel, HA, and laminin hydrogels show good biocompatibility with neural cells. However, they lack the printability property. These hydrogels have to be mixed with other printable hydrogels to enhance their printability. These printable bioinks do not show a flawless scaffold for neural cells to grow and differentiate. Therefore, neural biocompatible hydrogels with appropriate bioprinting properties have to be synthesized. It is likely that future progresses in material science will offer more flexibility to control cell adhesion and cytocompatibility with favorable mechanical properties and printability. Such bioprinted neural tissues hold great promise.

## Data Availability

The data that support the findings of this study are available from the corresponding authors upon reasonable request.
